# Case of Myelodysplastic Syndrome 15 Years After Kidney Transplantation Under Long‐Term Immunosuppression

**DOI:** 10.1155/crh/1471228

**Published:** 2026-07-03

**Authors:** Shuai Su, Sijia Yan, Liting Chen, Yi Xiao

**Affiliations:** ^1^ Department of Hematology, Tongji Hospital, Tongji Medical College, Huazhong University of Science and Technology, Wuhan, 430030, Hubei, China, hust.edu.cn

**Keywords:** kidney transplantation, myelodysplastic syndromes, pancytopenia

## Abstract

In solid organ transplant recipients receiving long‐term immunosuppression, persistent or progressive pancytopenia is often initially attributed to infections or drug toxicity, thereby potentially delaying the recognition of clonal myeloid disorders and germline predispositions. We report the case of a kidney transplant recipient who had predominantly maintained tacrolimus‐based immunosuppression and presented with progressive pancytopenia and marked reticulocytopenia. Bone marrow evaluation revealed severe hypocellularity with suppression of granulopoiesis and erythropoiesis, as well as prominent dysmegakaryopoiesis. Flow cytometry showed a small population of immunophenotypically aberrant myeloid blasts, supporting a diagnosis of hypocellular myelodysplastic syndrome. Myeloid gene next‐generation sequencing detected a FANCA missense variant (c.3630C > A; p.F1210L; variant allele frequency 47.2%), prompting consideration of germline‐associated marrow failure or genetic susceptibility and the need for confirmatory testing in nonhematopoietic tissues. During hospitalization, the patient developed severe opportunistic infections that rapidly progressed to respiratory failure and hemodynamic instability. This case highlights the need for early marrow evaluation and genetic risk stratification in transplant recipients with unexplained cytopenia and for the dynamic balancing of hematopoietic rescue against preservation of allograft function to reduce diagnostic delays and subsequent complications.

## 1. Introduction

Myelodysplastic syndrome (MDS) is extremely rare in long‐term survivors after kidney transplantation. Previous studies indicate that solid organ transplant recipients have an approximately 4‐fold higher relative risk of myeloid neoplasms than the general population; however, the 10‐year cumulative incidence post‐transplantation is only approximately 0.11%–0.13% [1]. In kidney transplant recipients, chronic immunosuppression and susceptibility to infection lead to a broad differential diagnosis of cytopenia. In addition, the clinical and morphological features of hypocellular MDS (HMDS) can overlap with those of bone marrow failure syndromes such as aplastic anemia (AA), further complicating the diagnosis [2]. Once diagnosed, the complex immune milieu frequently constrains treatment options, and outcomes are generally poor, with a median survival of approximately 1 year having been reported in transplant recipients with MDS [3].

Here, we describe a case of MDS that developed 15 years after kidney transplantation. We summarize the proposed mechanisms, the diagnostic evidence chain, and the clinical course, aiming to improve recognition of this rare scenario and to discuss key diagnostic and therapeutic challenges and potential strategies.

## 2. Case Presentation

A 61‐year‐old man underwent renal allograft transplantation in 2010 for uremia (donor information was unavailable). After transplantation, the patient received long‐term maintenance immunosuppression with oral tacrolimus plus mizoribine, with dose adjustments based on trough concentrations and regular clinical monitoring. Allograft function remained relatively stable, with serum creatinine fluctuating around 200 μmol/L. At a routine follow‐up in August 2024, the complete blood count was normal; serum creatinine was 189 μmol/L, estimated glomerular filtration rate (eGFR) was 32.5 mL/min/1.73 m^2^, and the tacrolimus trough concentration was 11 ng/mL. Computed tomography revealed massive ascities and tuberculous peritonitis. His condition improved after adjusting the immunosuppressant dose and initiating antituberculosis therapy.

On December 5, 2025, he was admitted with a 5‐day history of vomiting and diarrhea. Laboratory testing revealed white blood cell (WBC) count of 1.52 × 10^9^/L, absolute neutrophil count (ANC) of 0.83 × 10^9^/L, hemoglobin of 89 g/L, and platelet count of 18 × 10^9^/L. Alanine aminotransferase was 67 U/L, aspartate aminotransferase was 90 U/L, total protein was 49.1 g/L, albumin was 26.5 g/L, creatinine was 214 μmol/L, eGFR was 29.7 mL/min/1.73 m^2^, lipase was 125.1 IU/L, and amylase was 95 U/L. High‐sensitivity C‐reactive protein was 8.3 mg/L, and procalcitonin was 0.14 ng/mL. High‐sensitivity troponin I was 90.8 pg/mL, myoglobin was 203.2 ng/mL, creatine kinase‐MB was 6.9 ng/mL, and N‐terminal pro–B‐type natriuretic peptide was 4614.0 pg/mL. The urinary albumin‐to‐creatinine ratio was 253.5 μg/mg. The tacrolimus trough concentration was 4.1 ng/mL. EBV‐DNA, CMV‐DNA, BK virus DNA, JC virus DNA, and parvovirus B19 DNA were negative in peripheral blood. There was no clinical or laboratory evidence of chronic active EBV disease. HIV antigen/antibody testing was negative, and T‐SPOT.TB was also negative. Targeted capture next‐generation sequencing of blood pathogens did not detect HTLV‐1, HTLV‐2, *Mycobacterium tuberculosis* complex, or other organisms suggestive of disseminated infection. Standard serological testing for HTLV‐1/HTLV‐2 was not routinely available at our institution and was therefore not performed. Heavy‐metal screening showed values within the reference ranges. Urinalysis revealed protein 2+. The stool was yellow and loose; microscopy revealed red blood cells 2–6 per high‐power field (HPF), WBC “full field,” and a positive fecal occult blood test. Fungi were observed, and the stool culture grew *Saccharomyces cerevisiae*. Abdominal CT showed no new abnormalities. He had no other notable medical history. Vital signs were stable on admission, and the physical examination was unremarkable.

The initial working diagnoses included infectious diarrhea, pancytopenia of unclear etiology, and renal allograft dysfunction. He received antimicrobial therapy, transfusion support, and hematopoietic stimulation, followed by a hematologic evaluation. The reticulocyte count was 0.003 × 10^12^/L (0.11%). Ferritin was 1546.9 μg/L, with normal serum iron. Hemoglobin electrophoresis revealed elevated HbA_2_ (4.3%), but thalassemia genotyping was negative. Folate and vitamin B12 levels were within the normal range. Plasma‐free hemoglobin and Coombs test results were negative.

**FIGURE 1:**
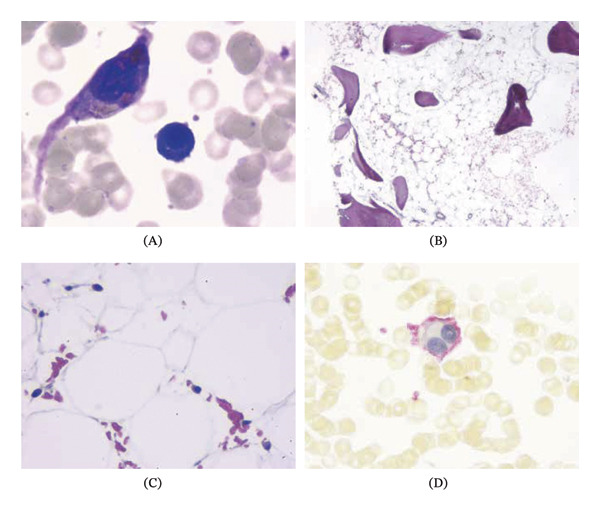
Results of bone marrow examinations. (A) Bone marrow aspirate smear showing markedly hypocellular marrow with rare platelets and small megakaryocytes, including mononucleated and binucleated forms, with granulocytic and erythroid suppression and relative lymphocytosis. (B and C) Bone marrow biopsy showing severely hypocellular marrow (cellularity < 10%), predominantly adipose tissue with a paucity of hematopoietic elements; scattered mature lymphocytes and plasma cells are present. (D) Micromegakaryocyte enzyme staining supporting megakaryocytic proliferation with dysplasia; 37 megakaryocytes were identified, including increased micromegakaryocytes (mononucleated, binucleated, and multinucleated) and lymphoid‐like micromegakaryocytes.

Bone marrow aspirate morphology showed markedly hypocellular marrow with suppressed granulopoiesis and erythropoiesis. Dysmegakaryopoiesis was prominent with small megakaryocytes, including mononucleated and binucleated forms. Bone marrow biopsy revealed severe hypocellularity (< 10%) with dysmegakaryopoiesis but did not show granulomatous inflammation, caseous necrosis, hemophagocytosis, or other histopathological findings suggestive of infectious marrow involvement. Specific bone marrow PCR, culture, or special staining for HIV or *Mycobacterium tuberculosis* was not performed because the available systemic and marrow findings did not suggest HIV‐ or tuberculosis‐related marrow involvement. Flow cytometry revealed approximately 0.5% myeloid blasts (of all nucleated cells) with an aberrant immunophenotype. The lymphocyte count did not increase, and the subset distribution was broadly normal, without a clear monoclonal lymphoid population. Neutrophils were decreased and exhibited an abnormal maturation pattern. Monocytes were not increased. Karyotype analysis yielded 46, XY [1]. Next‐generation sequencing detected a FANCA missense variant, NM_000135 (exon 37): c.3630C > A (p.F1210L), with a variant allele frequency of 47.2%. Based on these findings, a diagnosis of MDS was established (representative bone marrow findings are summarized in Figure 1).

**FIGURE 2:**
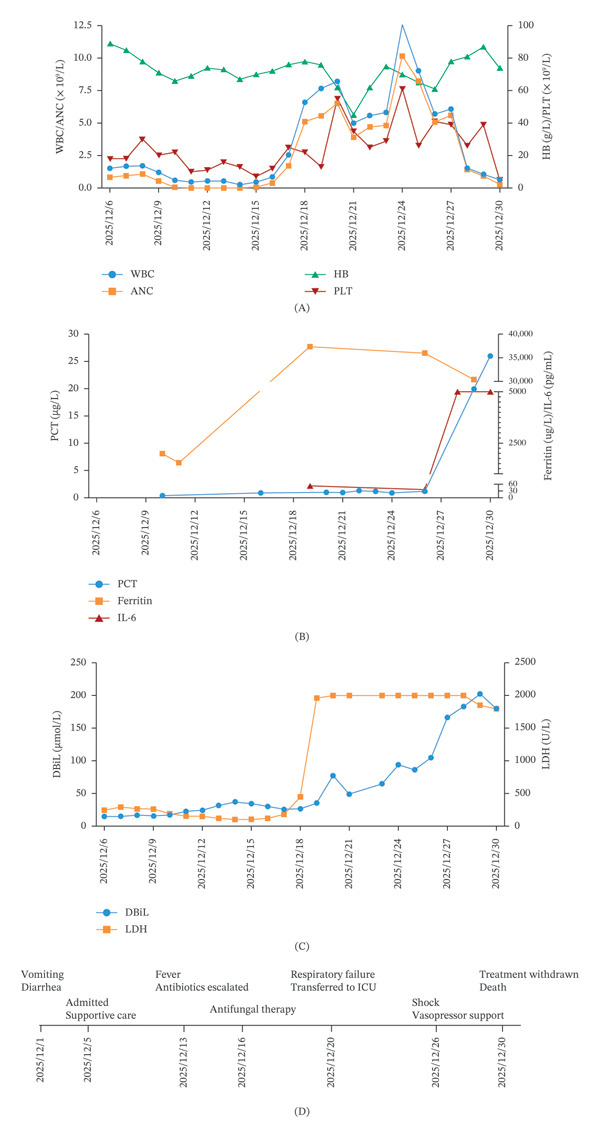
Dynamic changes in laboratory examinations and key clinical events (A) Serial complete blood counts showing trends in WBC, ANC, Hb, and PLT. (B) Time course of inflammatory and immune activation markers, including PCT, ferritin, and IL‐6. (C) Liver injury/hemolysis‐associated markers, including DBiL and LDH. (D) Timeline of major clinical events. Abbreviations: WBC, white blood cell; ANC, absolute neutrophil count; Hb, hemoglobin; PLT, platelet count; PCT, procalcitonin; IL‐6, interleukin‐6; DBiL, direct bilirubin; LDH, lactate dehydrogenase; ICU, intensive care unit.

During hospitalization, the WBC count transiently improved following transfusion and hematopoietic stimulation, whereas hemoglobin and platelet counts remained persistently low. From December 6 to December 30, 2025, the patient required repeated transfusion support, including platelet transfusions on 18 separate days with a cumulative dose of 22 units and red blood cell transfusions on 8 separate days with a cumulative dose of 21.5 units. The transfusion requirement increased during the later clinical course, particularly after December 19, when frequent platelet transfusions and repeated red blood cell transfusions were required. Despite intensive transfusion support and hematopoietic stimulation, platelet and hemoglobin recovery was not sustained. On December 30, the complete blood count still showed severe pancytopenia, with WBC of 0.64 × 10^9^/L, ANC of 0.28 × 10^9^/L, hemoglobin of 74 g/L, and platelet count of 5 × 10^9^/L. On December 13, 2025, the patient developed fever and oxygen desaturation, and chest CT findings suggested a pulmonary infection. Next‐generation blood metagenomic sequencing detected *Aspergillus flavus* complex and *Rhizomucor pusillus*. Antibiotic therapy was escalated, and antifungal therapy was initiated. Despite treatment, the temperature and oxygenation fluctuated. On December 20, 2025, he developed respiratory failure and was transferred to the intensive care unit for endotracheal intubation and mechanical ventilation. Supportive measures included bronchoscopy with bronchoalveolar lavage, prone positioning, anti‐infective therapy, transfusion support, and symptomatic care. The patient’s condition continued to deteriorate and was complicated by septic shock. On December 30, 2025, life‐sustaining treatment was withdrawn at the family’s request, and the patient died thereafter. Figure 2 summarizes the inpatient course and test results.

## 3. Discussion

In solid organ transplant recipients receiving long‐term immunosuppression who present with severe pancytopenia, cytopenia is often initially attributed to infection or drug toxicity, potentially delaying the recognition of clonal myeloid diseases. In this case, key features included the rapid onset of profound trilineage cytopenia after acute gastrointestinal symptoms, extreme reticulocytopenia, and marked hypocellular marrow, followed by rapid progression to fatal infection. These findings suggest that, when marrow failure in transplant recipients overlaps with a clonal process, the clinical course may deteriorate abruptly.

Differential diagnoses can be organized into three axes: (i) drug‐ and transplant‐related marrow suppression; (ii) potentially reversible suppression due to infection or nutritional deficiency; and (iii) distinction between AA and HMDS. In this patient, there was insufficient evidence for reversible suppression due to infection or nutritional deficiency. Active parvovirus B19, Epstein–Barr virus, and cytomegalovirus infection were not supported by the available virological evaluation. HIV antigen/antibody testing was negative. Standard serological testing for HTLV‐1/HTLV‐2 was not performed because it was not routinely available at our institution; however, targeted capture next‐generation sequencing of blood pathogens did not detect HTLV‐1 or HTLV‐2. Active or disseminated tuberculosis was not supported by a negative T‐SPOT. TB result and negative targeted capture next‐generation sequencing for blood pathogens, which did not detect *Mycobacterium tuberculosis* complex. Brucellosis was not supported by the clinical history, epidemiological exposure history, or blood pathogen sequencing. Folate and vitamin B12 levels were within the normal range, and thalassemia genotyping was negative. Heavy metal testing showed values within the normal reference ranges, and there was no evidence of clinically relevant heavy metal exposure or chronic alcohol use. Serum copper and ceruloplasmin levels were within the normal ranges. The principal diagnostic consideration was whether the patient had AA or HMDS. Both entities can present with hypocellular marrow and severe pancytopenia and may be precipitated by infectious triggers. However, marrow morphology demonstrated dysmegakaryopoiesis; flow cytometry revealed abnormal granulocytic maturation and an immunophenotypically aberrant myeloid blast population; and molecular testing identified a clonal abnormality (a high‐burden FANCA variant). Although the reported karyotype was normal, only a single metaphase was observed; therefore, cytogenetic abnormalities could not be excluded. Taken together, these findings support a diagnosis of HMDS rather than AA.

Accumulated evidence suggests that myeloid neoplasms after solid organ transplantation are unlikely to result from a single factor. Instead, they may reflect the combined effects of long‐term drug exposure, which promotes mutagenesis and clonal selection; impaired immune surveillance that permits clonal escape; and hematopoietic stress driven by chronic inflammation, infection, and antigenic stimulation [4].

Immunosuppressants may drive clonal evolution through mutagenesis and selective expansion. Cohort studies have reported a dose–response association between purine analogs, such as azathioprine, and the increased risk of post‐transplant myeloid neoplasms. Azathioprine may preferentially expand hematopoietic cells with DNA repair defects in certain genetic backgrounds, acting as both a DNA‐damaging agent and a selective pressure during clonal evolution [5]. Although the patient did not receive azathioprine, he had prolonged exposure to mizoribine, a purine synthesis inhibitor. In kidney transplant studies, hemoglobin, WBC, and platelet indices are commonly monitored as safety endpoints after mizoribine administration. A prospective randomized trial suggested an association between mizoribine and leukopenia, supporting the need for close monitoring [6]. Tacrolimus, a calcineurin inhibitor, is classically associated with nephrotoxicity and neurotoxicity [7]; however, case reports have described reversible cytopenia or marrow changes that resolve after drug discontinuation or dose adjustment [8]. Thus, this case may reflect cumulative hematopoietic stem cell injury due to long‐term immunosuppressive exposure, with chronic stress providing a selective advantage for abnormal clones.

Impaired immune surveillance may also contribute to clonal escape after transplantation. Chronic iatrogenic immunosuppression reduces the clearance of mutant cells, allowing the expansion of abnormal hematopoietic clones that may otherwise remain constrained. In the general population, clonal hematopoiesis with mutations in genes such as DNMT3A and TET2 increases with age; however, most individuals do not develop a hematologic malignancy, likely due to clonal competition, immune surveillance, and microenvironmental constraints [9]. Long‐term exposure to tacrolimus and glucocorticoids, which suppress T cell function, may weaken the control of latent clones and promote clonal evolution [10, 11]. Morton et al. reported that the incidence of myeloid neoplasms after transplantation may correlate with immunosuppressive intensity, with a higher risk in heart–lung transplant recipients than in kidney transplant recipients [1]. Immune deficiency is likely to provide a permissive background rather than a sufficient cause and must interact with clonal driver events to induce disease [12, 13]. In this case, cytogenetic evidence was limited by analysis of a single metaphase; however, the presence of a high‐burden FANCA variant suggests the emergence of a competitively advantaged clone under weakened immune surveillance, which may have contributed to the rapid clinical decline.

Chronic antigenic stimulation, opportunistic infections, and inflammatory stress may shape the marrow microenvironment and intensify clonal selection. Persistent exposure to donor antigens may sustain low‐grade inflammation and regenerative pressure despite immunosuppression. Donor antigen stimulation and opportunistic infections have been proposed to drive stress hematopoiesis, thereby increasing the likelihood of acquiring key genetic alterations [14]. In the current patient, prolonged immunosuppression and prior infections (including tuberculous peritonitis) likely created sustained hematopoietic stress; the high‐burden FANCA variant is consistent with the establishment of an advantageous abnormal clone under chronic pressure.

Myeloid next‐generation sequencing revealed a FANCA variant (variant allele frequency, 47.2%), raising two major possibilities: (i) a germline predisposition–related marrow failure spectrum with progression toward MDS [15] or (ii) acquired clonal evolution in MDS promoted by long‐term immunosuppression and chronic post‐transplant stress. A variant allele frequency near 50% is compatible with a heterozygous germline variant or a dominant heterozygous somatic clone; definitive distinction requires testing of nonhematopoietic tissue. Longitudinal sequencing was not available; therefore, we could not determine whether the FANCA variant remained stable over time or showed evidence of clonal expansion. Because of resource constraints, fluorescence in situ hybridization for MDS‐associated abnormalities and comprehensive germline evaluation were not feasible. Nevertheless, the diagnostic evidence supporting MDS—integrating morphology, flow cytometric abnormalities, and a clonal variant—remains compelling.

The management of MDS in transplant recipients requires balancing hematopoietic improvement with the maintenance of allograft function [16]. In the present report, a 61‐year‐old kidney transplant recipient required continued immunosuppression to preserve renal allograft function. From a curative standpoint, allogeneic hematopoietic stem cell transplantation (HSCT) could theoretically control disease; however, HSCT after solid organ transplantation faces several practical barriers: (i) immunosuppression often must be modified or discontinued to ensure engraftment, increasing the risk of acute allograft rejection; (ii) conditioning and immune reconstitution increase the risks of infection and organ injury; and (iii) donor availability and HLA‐matching constraints make same‐donor transplantation impractical in most cases. Consequently, HSCT is not feasible within the short clinical window. For non‐transplantation approaches, low‐intensity regimens and supportive care should be considered [17]. Hypomethylating agents typically require multiple cycles for a response, and a substantial proportion of patients fail to respond. A meaningful treatment window may be absent in patients with profound hypocellular marrow, severe baseline pancytopenia, or rapid progression to severe infection. Immunosuppressive therapy for HMDS can render approximately 30% of patients transfusion‐independent and induce complete remission in 11% of patients [18]. However, our patient developed progressive marrow failure despite ongoing tacrolimus exposure, making the benefit of intensified immunosuppression unlikely and potentially increasing infection risk.

Supportive care (transfusion, hematopoietic stimulation, and intensified anti‐infective therapy) was the only feasible bridging strategy; however, the patient rapidly progressed to severe pneumonia, respiratory failure, and septic shock. This underscores that infection may become the earliest and most decisive determinant of outcomes in transplant recipients facing profound neutropenia and pharmacological immunosuppression.

## 4. Conclusion

During long‐term follow‐up after kidney transplantation, the development of unexplained bilineage or trilineage cytopenia should prompt a strong suspicion of an underlying bone marrow failure disorder. Early bone marrow evaluation is essential for establishing a diagnosis and avoiding missed opportunities to exploit a potential therapeutic window. Under ongoing immunosuppression, the differentiation of hypoplastic/hypocellular MDS requires particular emphasis on evidence of clonality. An integrated assessment incorporating bone marrow morphology, flow cytometric immunophenotyping, and molecular testing is critical for distinguishing MDS from AA and from marrow suppression due to drugs, infection, or other reversible causes.

Management of MDS in transplant recipients requires an individualized approach. While preserving allograft function, clinicians should consider disease‐modifying approaches early to reduce the risk of progression and, when feasible, assess candidacy for HSCT. Multidisciplinary management and proactive supportive care are central to improving outcomes in this high‐risk population.

## Author Contributions

Shuai Su collected the clinical data and drafted the manuscript. Sijia Yan contributed to data organization and manuscript revision. Liting Chen reviewed and edited the manuscript. Yi Xiao acquired funding, reviewed, and edited the manuscript.

## Funding

The work was funded by grants from National Natural Science Foundation of China (nos. 81873444, 82070213, and 82370196 to Yi Xiao).

## Disclosure

All authors have read and approved the final version of the manuscript. Yi Xiao had full access to all of the data in this study and takes complete responsibility for the integrity of the data and the accuracy of the data analysis.

## Consent

Written informed consent for publication of the patient’s clinical information and related medical images was obtained from the patient’s family.

## Conflicts of Interest

The authors declare no conflicts of interest.
